# Bidirectional Tachycardia after an Acute Intravenous Administration of Digitalis for a Suicidal Gesture

**DOI:** 10.1155/2014/109167

**Published:** 2014-08-24

**Authors:** Diletta Sabatini, Giovanni Truscelli, Antonio Ciccaglioni, Carlo Gaudio, Maria Caterina Grassi

**Affiliations:** ^1^Emergency Toxicology and Poison Control Center Unit, Policlinico Umberto I and Department of Physiology and Pharmacology “V. Erspamer”, School of Medicine, Sapienza University of Rome, Piazzale Aldo Moro 5, 00185 Rome, Italy; ^2^Department of Cardiovascular, Respiratory, Nephrological, Anesthesiological, and Geriatric Sciences, Policlinico Umberto I, Sapienza, University of Rome, Viale del Policlinico 155, 00161 Rome, Italy

## Abstract

Acute digoxin intoxication is a life-threating condition associated with severe cardiotoxicity. Female gender, age, low lean body mass, hypertension, and renal insufficiency may worsen the prognosis. Arrhythmias caused by digitalis glycosides are characterized by an increased automaticity coupled with concomitant conduction delay. Bidirectional tachycardia is pathognomonic of digoxin intoxication, but it is rarely observed. An 83-year-old woman was admitted to the Emergency Department after self-administration of 5 mg of digoxin i.v. for suicidal purpose. Her digoxin serum concentration was 17.4 ng/mL. The patient developed a bidirectional tachycardia and the Poison Control Center of the hospital provided digoxin immune fab. Bidirectional tachycardia quickly reversed and the patient remained stable throughout the hospital stay. This case shows that a multiple disciplinary approach, involving cardiologists and toxicologists, is essential for the management of digoxin intoxication. The optimal treatment of this rare event depends on the clinical conditions and on the serum drug concentration of the patient. Digoxin immune fab represents a safe, effective, and specific method for rapidly reversing digitalis cardiotoxicity and should be started as soon as the diagnosis is defined.

## 1. Introduction

Nowadays, digitalis intoxications are rarely observed because the indications for digoxin administration are limited to advanced heart failure and atrial fibrillation. Intoxication can occur during chronic treatment with digoxin or acutely following a massive intake of the drug. Chronic digitalis intoxications are common in elderly and in certain clinical conditions [1, 2]. However, digitalis intoxication remains a challenging diagnosis since symptoms and electrocardiographic (ECG) abnormalities caused by cardiac glycosides are not specific [3]. Bidirectional tachycardia (BT), consisting in beat-to-beat alternation of morphology and axis of QRS complexes, may be pathognomonic, but this is uncommon [4].

Digitalis acute intoxications may be observed in suicidal patients [5, 6]. Although suicidal attempts by acute ingestion of many tablets are reported in the literature, there are no cases concerning acute intravenous digitalis overdose. Hereafter, we describe a case of acute digitalis intoxication after intravenous administration for suicidal purpose, with the subsequent development of BT.

## 2. Case Details

An 83-year-old woman was admitted to the Emergency Department (ED) of Umberto I, Policlinico of Rome for an acute intravenous (i.v.) self-administration of digoxin. The patient self-injected 5 mg of digoxin i.v. (Lanoxin 10 vials; 0.5 mg/2 mL each) for suicidal attempt three hours prior to hospital admission. She informed, through a telephone call, her sister about the suicidal attempt and hence the ED was alerted.

On admission, the patient was fully conscious (Glasgow Coma Scale: 15) and on her left forearm, near the antecubital area, signs of extravasation were present caused by a direct leakage from a mispositioned venous access. A first electrocardiogram (ECG) demonstrated sinus rhythm.

The patient was afebrile, and her pulse and respiratory rates were 90 beats/min and 18 breaths/min, respectively. Blood pressure was 110/70 mm Hg and no peripheral edema was present. Auscultation revealed regular rhythm with no murmurs, rubs, or gallops. She had decreased breath sounds in her posterior lung bases. The patient was overweight (BMI 31.2 kg/m^2^) and had a previous history of insomnia and depressed mood, for which reason she was self-administering Fluoxetine for several years. Moreover, she was suffering from hypertension, and she was treated with angiotensin-converting-enzyme inhibitors and beta-blockers. Laboratory findings showed serum digoxin levels of 13.9 ng/mL (range 0.6–2.6 ng/mL), assessed through enzyme immunoassay technique [2]. All other parameters were within the range of normality, including potassium (4.15 mEq/L) and creatinine (0.7 mg/dL). Fluids and diuretics (0.9% sodium chloride 5000 mL at 100 mL/h infusion rate and furosemide 20 mg/2 mL, 1 vial) were administered.

Two hours after admission, an ECG exam was performed, showing a severe bradyarrhythmia that was subsequently treated with atropine (0.5 mg i.v.) followed by transcutaneous pacing to increase the heart rate of the patient [5, 7, 8].

**Figure 1:**
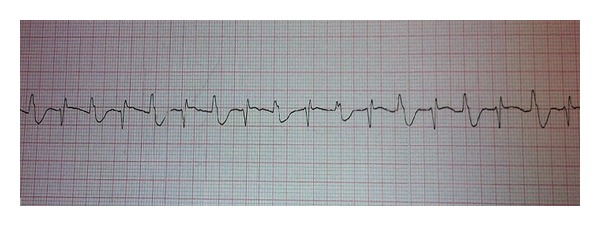
II lead ECG, in the Emergency Department, showing bidirectional tachycardia.

Three hours after admission, a BT developed and the blood pressure decreased to 90/60 mm Hg (Figure 1). The patient was promptly sedated with Sufentanil (250 mcg) and Fentanyl (200 mcg) to allow orotracheal intubation (IOT) and nasogastric tube (NG) placing. Since BT is usually associated with digitalis toxicity [4], the Poison Control Center of the hospital proposed to administer digoxin immune fab (DigiFab) and, according to the steady state concentration of digoxin, 440 mg was infused. The administration of DigiFab immediately restored normal sinus rhythm; the blood pressure increased (120/80 mm Hg) and five hours after admission IOT was removed. Twelve hours after admission, the digoxin serum level raised up to 17.4 ng/mL and thirty-six hours later declined to 7 ng/mL. Serum potassium level remained within the range (4.15 mEq/L at admission and 3.80 mEq/L forty-eight hours later).

The patient remained neurologically intact and additional ECG showed periods of various atrioventricular blocks with accelerated junctional rhythm (pulse rate: 60 beats/min) followed by episode of sinus bradycardia (40 beats/min). Seventy hours after admission to the ED, the patient was moved to the coronary care unit and remained stable throughout the hospital stay.

Thirteen days later, the patient was moved to the Psychiatric Department of the same hospital and was discharged after five days.

## 3. Discussion

Acute digoxin intoxication is a life-threatening condition associated with severe cardiotoxicity. Female gender, hypertension, and age may worsen the prognosis. Furthermore, digoxin half-life (range 36–51 hours) may be prolonged in overdose (72–94 hours) [5, 9]. In the case we are reporting, digoxin absorption was reduced by the extravascular leakage at the site of injection. Probably, extravascular leakage of the injected digoxin played a key role in the positive outcome of this patient. Moreover, the patient did not have an impaired renal function and her body weight was not low. Digoxin immune fab represents a safe, effective, and specific method of rapidly reversing digitalis cardiotoxicity, and treatment should be started as soon as diagnosis is defined.

In the current case BT developed as a consequence of digoxin intoxication. Digoxin cardiotoxicity is characterized by an increased automaticity coupled with concomitant conduction delay [8]. Although the development of BT following digoxin intoxication is uncommon and its mechanism remains unclear, this ECG abnormality is quite specific for digoxin cardiotoxicity.

In addition, as digitalis inhibits the sodium-potassium adenosine triphosphate pump leading to intracellular calcium overload, BT could be related to drug-induced triggered activity [10]. In this frame, diagnosis of digitalis cardiotoxicity represents a difficult challenge and requires a careful correlation of the ECG findings, patient medical history, physical examination, and laboratory analysis. As shown in this case report, a multiple disciplinary approach, involving cardiologists and toxicologists, is essential for the management of digoxin intoxication and resulting cardiotoxicity.
